# The Role of Artificial Intelligence in the Detection of Cardiac Amyloidosis: A Systematic Review

**DOI:** 10.7759/cureus.78488

**Published:** 2025-02-04

**Authors:** Fatima Ibrahim Abdalla Ibrahim, Mozdaher Gaffer Hussen Ali, Mohammed Hassan Awad Ali, Almontasir Belah Alsadig Abdalwahab Abdallah, Nisreen Galaleldin Elnoor Mohammed, Ammar Elhaj, Samir Ibrahim, Wadah Ahmed Osman Ahmed

**Affiliations:** 1 Internal Medicine, Najran Armed Forces Hospital, Ministry of Defense Health Services, Najran, SAU; 2 Pediatrics, Maternity and Children Hospital Najran, Najran, SAU; 3 Internal Medicine, University Hospital Waterford, Waterford, IRL; 4 Internal Medicine, Mullingar Hospital, Mullingar, IRL; 5 Internal Medicine, Kalba Hospital, Sharjah, ARE

**Keywords:** artificial intelligence, cardiac amyloidosis, echocardiography, electrocardiogram, machine learning

## Abstract

Cardiac amyloidosis (CA) is an underdiagnosed condition that occurs when misfolded amyloid proteins deposit in cardiac tissue causing progressive myocardial dysfunction. Artificial intelligence (AI) technology demonstrates strong potential to enhance the identification of various cardiovascular diseases, including CA. This systematic review examines how AI systems identify CA. This study explores AI's application as a diagnostic tool for CA.

We searched five databases (PubMed/MEDLINE, Scopus, Web of Science, Embase, and IEEE Xplore) for relevant studies using the Preferred Reporting Items for Systematic Reviews and Meta-Analyses (PRISMA) guidelines. Ten of the 629 studies we discovered were determined to be suitable for the current systematic review. We used the PICOS (P: population, I: intervention or exposure, C: comparison, O: outcome, S: study type) framework to collect relevant data from the included studies. The screening procedure was blinded and used predefined inclusion and exclusion criteria. It was undertaken in two phases. Whenever disagreements emerged, the reviewers deliberated and concluded.

After the screening procedure, 10 studies in all were judged suitable for this review. Through the analysis of standard laboratory data, transthoracic echocardiography, electrocardiogram, medical records, cardiac magnetic resonance, and whole-body scintigraphy, these studies assessed the potential usefulness of AI models in diagnosing CA.

AI models have been useful as a CA diagnostic tool, outperforming professional cardiologists in one instance or being on par with them in others.

## Introduction and background

Cardiac amyloidosis (CA) is a progressive and life-threatening condition characterized by the extracellular deposition of misfolded amyloid proteins in the myocardium, leading to restrictive cardiomyopathy, heart failure, and arrhythmias [1]. Historically considered rare, CA is now recognized as underdiagnosed, with prevalence estimates rising sharply among older adults and those with heart failure with preserved ejection fraction (HFpEF) [2]. While advancements in non-invasive imaging, such as cardiovascular magnetic resonance (CMR) and bone scintigraphy, have improved diagnostic accuracy, early detection remains elusive. Clinicians face a perfect storm of challenges, including heterogeneous symptoms mimicking common conditions, reliance on invasive biopsies for confirmation, and the limited specificity of traditional biomarkers - all contributing to diagnostic delays that forfeit opportunities for life-extending therapies [3,4].

This critical gap in timely diagnosis has ignited interest in artificial intelligence (AI) as a transformative solution. AI, encompassing machine learning (ML) and deep learning (DL), offers unparalleled capacity to analyze complex, multi-modal datasets, from electrocardiograms (ECGs) and echocardiography to serum biomarkers, uncovering subtle patterns invisible to the human eye [5,6]. For example, AI models trained on routine ECGs detect CA with C-statistics of 0.85-0.91, while echocardiography-based algorithms achieve near-perfect accuracy (C-statistics up to 1.00) in differentiating CA from other causes of cardiac hypertrophy [7]. These tools not only mitigate cognitive biases and inter-observer variability but also democratize expertise, enabling early detection in primary care or resource-limited settings where advanced imaging is unavailable [8]. Furthermore, AI’s ability to integrate disparate data, such as coagulation profiles or free light chains, into diagnostic signatures with >90% sensitivity/specificity could revolutionize screening, particularly for high-risk populations like elderly HFpEF patients [8]. Such innovations are urgently needed, as early diagnosis enables timely initiation of disease-modifying therapies (e.g., tafamidis for transthyretin amyloidosis), which can double survival in some cohorts [9].

Despite this promise, the field lacks a comprehensive synthesis of AI’s capabilities and limitations in CA detection. This systematic review evaluates AI’s performance across diagnostic modalities (ECG, echocardiography, CMR, and laboratory integration), highlighting its potential to address disparities in care through cost-effective screening and reduced reliance on invasive procedures. By critically appraising existing evidence, we aim to guide future research, optimize clinical workflows, and accelerate AI’s translation into a tool that redefines outcomes for patients with this underrecognized, deadly disease.

## Review

Methodology

Protocol

This systematic review was conducted according to the Preferred Reporting Items for Systematic Reviews and Meta-Analyses (PRISMA) 2020 guidelines [10]. Prior to data collection, a predefined protocol outlining objectives, inclusion/exclusion criteria, and analytical methods was developed. While the protocol was not registered in a public registry due to the exploratory scope of the review, which required iterative refinements to adapt to the rapidly evolving and heterogeneous landscape of AI applications in CA detection, all deviations from the initial plan were documented transparently.

Eligibility Criteria

The eligibility criteria were structured using the PICOS (population, intervention or exposure, comparison, outcome, and study type) framework [11]. The population (P) included adults (≥18 years) diagnosed or suspected of CA, excluding pediatric or non-CA studies. The intervention (I) involved AI technologies (e.g., ML, DL, and natural language processing) for CA detection or diagnosis, excluding non-diagnostic applications. Comparisons (C) were made against traditional diagnostic methods or other AI techniques, excluding studies without validation against a reference standard. Outcomes (O) included diagnostic performance metrics (e.g., accuracy, sensitivity, specificity, and area under the curve (AUC)), excluding non-diagnostic outcomes. Eligible study designs (S) were original research (cross-sectional, cohort, case-control, diagnostic accuracy), excluding reviews, editorials, case reports, and non-peer-reviewed studies (Table 1).

**Table 1:** Inclusion and exclusion criteria based on the PICOS framework. PICOS: population, intervention or exposure, comparison, outcome, and study type.

PICOS element	Inclusion criteria	Exclusion criteria
Population	Patients diagnosed or suspected of having cardiac amyloidosis.	Studies focusing on conditions other than cardiac amyloidosis.
Intervention	Application of artificial intelligence (AI) technologies for the detection or diagnosis of cardiac amyloidosis.	Studies not utilizing AI technologies for cardiac amyloidosis detection.
Comparison	Traditional diagnostic methods or other AI techniques.	Studies without comparison to traditional or alternative AI methods.
Outcomes	Diagnostic performance metrics such as accuracy, sensitivity, and specificity.	Studies lacking data on diagnostic performance metrics.
Study design	Original research articles, including cross-sectional, cohort, case-control, and diagnostic accuracy studies.	Review articles, editorials, case reports, and conference abstracts.
Language	Peer-reviewed articles published in English.	Non-English publications.
Access	Availability of full-text articles.	Studies without full-text access.

Search Strategy

A comprehensive search was conducted on PubMed/MEDLINE, Scopus, Web of Science, Embase, and IEEE Xplore to identify relevant studies. The search strategy was designed using a combination of Medical Subject Headings (MeSH) terms, keywords, and Boolean operators. The reference lists of included studies were manually reviewed to identify any additional articles relevant to the review. Each search string was adapted to the specific requirements and indexing terms of the respective databases. The detailed search strings for each database are provided in the Appendix.

Study Selection

All identified studies were imported into EndNote X9.3.3 (Bld 13966; Clarivate, Philadelphia, PA), and duplicates were removed while extracting the same from different databases. Two independent reviewers from the author's list screened the titles and abstracts for relevance. Studies that have full-text articles available were assessed as per inclusion and exclusion criteria. Disagreements among the first two reviewers were resolved through a third reviewer (tiebreaker).

Data Extraction

A standardized Microsoft® Excel spreadsheet (Microsoft Corporation, Redmond, WA) was used to collect the data from included studies. The extracted data encompassed study characteristics such as author, publication year, study design, and setting. Details about the population were recorded, including sample size, demographics, and clinical features. Information on AI methodology was captured, focusing on the type of AI algorithm used, as well as the training, validation, and testing methods applied. Additionally, outcomes were documented, including diagnostic accuracy, sensitivity, specificity, and other performance metrics.

Risk of Bias Assessment

The risk of bias was assessed using the QUADAS-2 (Quality Assessment of Diagnostic Accuracy Studies) tool. Each study was evaluated for potential bias in four domains: patient selection, index test, reference standard, and flow and timing.

Results

Search Results

**Figure 1:**
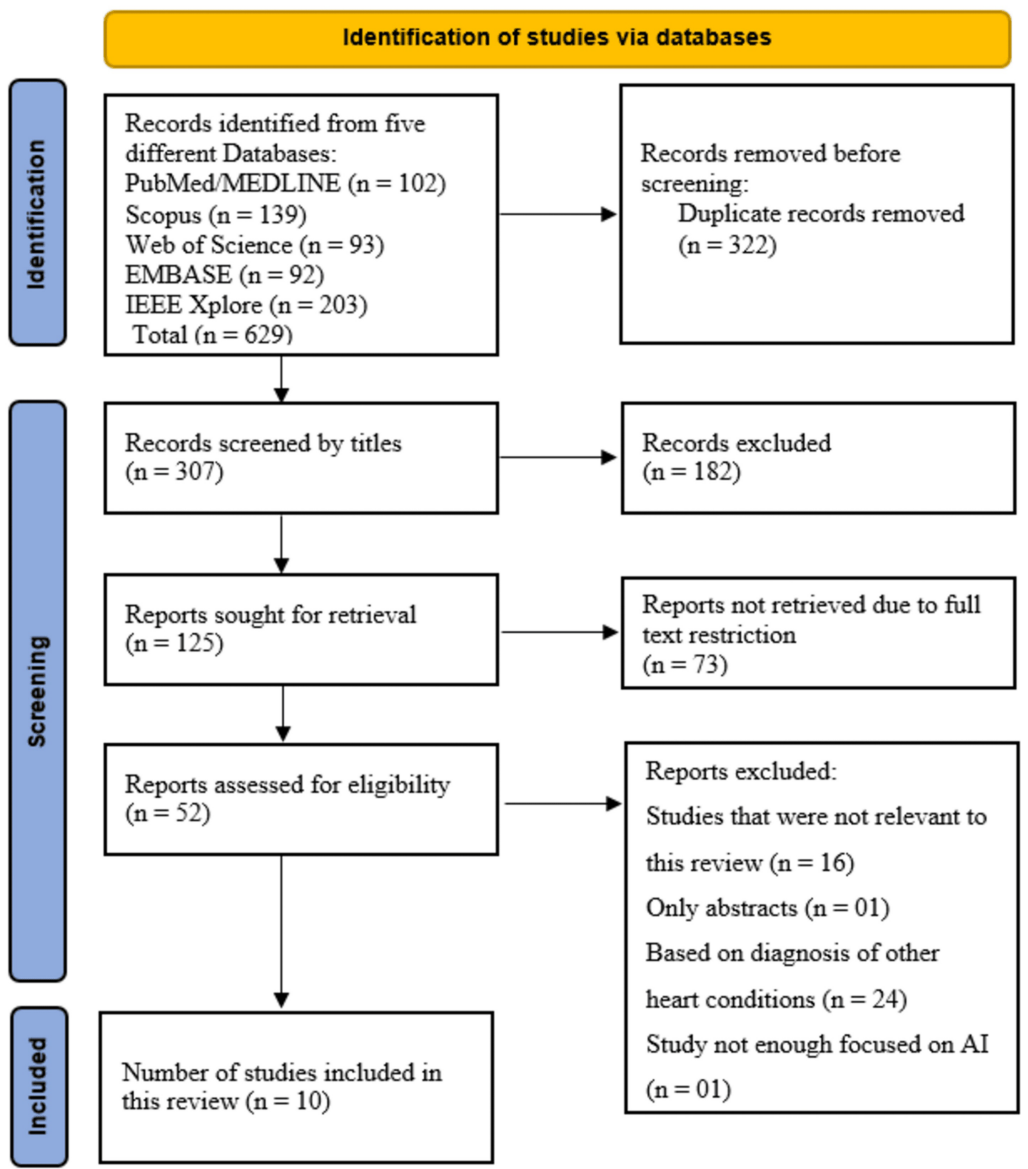
PRISMA (Preferred Reporting Items for Systematic Reviews and Meta-Analyses) flowchart.

On these databases, we found 629 distinct studies. A total of 322 studies were removed as duplicates after all of the studies were extracted and added to the EndNote library. After reviewing the titles of the remaining 307 papers, 182 titles that were unrelated to our study were eliminated. We looked at the remaining 125 studies to determine if the full-text papers were accessible. Because of restrictions on open access, 73 of these studies were excluded. Finally, 42 full-text papers were eliminated out of the remaining 52; 24 were about other heart disorders, one study had only an abstract, and 16 were unrelated to this review. Ten full-text papers were included in this systematic review (Figure 1).

Characteristics of Included Studies

The studies included in this systematic review showed that several forms of AI, including ML, convolutional neural network (CNN), and DL, could potentially be used in a variety of diagnostic procedures, including ECG, laboratory parameters, transthoracic echocardiogram (TTE), CMR, bone scintigraphy, as well as medical and nursing data (Table 2).

**Table 2:** Characteristics and key outcomes of studies included in this systematic review. ECG: electrocardiography; Echo: echocardiography; CA: cardiac amyloidosis; AI: artificial intelligence; ML: machine learning; DL: deep learning; CMR: cardiac magnetic resonance imaging; WBS: whole-body scintigraphy; PPV: positive predictive value; AUC: area under the curve; ROC: receiver operating characteristic; CNN: convolutional neural network; GLS: global longitudinal strain; LVEF: left ventricular ejection fraction; HCM: hypertrophic cardiomyopathy; CTRL: control group; HF: heart failure; SVM: support vector machine; RBF: radial basis function.

Author and publishing year	Study objective	Study design	Sample size	Type of AI	Diagnostic method for CA	Outcomes
Goto et al., 2021 [12]	The prospect of artificial intelligence in accurately identifying cardiac amyloidosis using ECG and echocardiography	Retrospective study	Echo group: patients = 609; controls = 3303. ECG group: patients = 597; controls = 8612	Machine learning	Echo and ECG	AI improves CA diagnosis. ECG ML model PPV: 3–4% (52–71% recall). Echo model recall: 67% (pre-screened by ECG), PPV: 33% → 77%.
Martini et al., 2020 [13]	Possible application of DL techniques for CA diagnosis using CMR	Randomized control trial	Patients = 107; control = 99	Deep learning and machine learning	CMR	DL achieved AUC: 0.982, accuracy: 88%, recall: 95%, precision: 83%, F1-score: 89%. ML model AUC: 0.952 (p = 0.39).
Grogan et al., 2021 [14]	Potential use of AI to detect CA from ECG data	Retrospective study	Patients = 508; controls = 491	Deep neural network	ECG	AI predicted CA with AUC: 0.91, PPV: 0.86. Detected CA in 60% of cases before clinical diagnosis (with repeated ECGs).
Delbarre et al., 2023 [15]	Using large hospital databases to design a CNN that can detect significant myocardial uptake on WBS.	Retrospective study	1633 WBS images	CNN	WBS	CNN identified CA. Cross-validation: AUC: 0.999, specificity: 99.5%, sensitivity: 98.9%. External validation: AUC: 0.999, specificity: 99.5%, sensitivity: 96.1%.
Cotella et al., 2023 [16]	Assessment of the possible AI application for LVEF as well as GLS computation in CA patients.	Retrospective validation study	Total sample = 51	Machine learning	Echocardiography	AI accurately computed GLS and LVEF. Sensitivity/specificity: pre-CA (83%/86%), post-CA (70%/79%).
Eckstein et al., 2022 [17]	Potential use of machine learning to identify CA based on cardiac function and bi-atrial and right ventricular strain.	Retrospective, single-center, observational design	CTRL = 44, HCM = 20, CA = 43	Machine learning	CMR	ML detected CA with 82% accuracy (top 10 variables) and 90.9% (41 variables). SVM RBF: sensitivity: 100%, accuracy: 94%, F1-score: 97%.
Duffy et al., 2022 [18]	Finding the cause of the increased left ventricle wall thickness, whether it is CA or hypertrophy, using DL.	Retrospective cross-sectional cohort	23,745 patients	Deep learning	Echocardiography	DL detected CA and hypertrophic disease. AUC: CA (0.79), hypertrophic disease (0.89).
García-García et al., 2021 [19]	ML as a diagnostic tool for CA.	Retrospective, transversal, descriptive, observational study	HF = 2861, CA = 27	Machine learning	Nursing and medical data acquired while a patient is in the hospital	ML specificity: 0.96, sensitivity: 0.56 for CA detection in HF/stroke patients. ROC AUC: 0.88.
Agibetov et al., 2021 [20]	The ability of a CNN-based artificial intelligence model to diagnose CA by examining CMR.	Retrospective and observational design	CA = 420, HF = 82	CNN	CMR	CNN diagnosed CA from CMR. Fine-tuned CNN: AUC: 0.96.
Agibetov et al., 2020 [21]	The possible use of AI for CA diagnosis based on standard laboratory parameters.	Prospective single-center study	CA = 124, HF = 36	Machine learning	Lab parameters	Best ML model (gradient-boosted trees) AUC: 0.86, sensitivity: 89.2%, specificity: 78.2%. ML distinguished CA-related HF from non-CA HF.

Summary of Diagnostic Performance Metrics

Agibetov et al.'s [20] laboratory parameters model had a receiver operating characteristic (ROC) AUC of 0.86 and specificity and sensitivity of 78.2% and 89.2%, respectively. With an ROC AUC of 0.88, the medical as well as nursing records ML showed a specificity and sensitivity of 96% and 56%, respectively. Out of all the TTE models, the ML model that Cotella et al. [16] developed to detect aberrant left ventricular ejection fraction (LVEF) had a specificity and sensitivity of 86% and 83% while the TTE was conducted before CA, and 70% and 79% when CA was diagnosed. Duffy et al. [18] created a TTE-based DL model that showed an ROC AUC of 0.79. Additionally, Goto et al.'s [12] integrated ECG-TTE ML demonstrated a 74-77% positive predictive value (PPV). Grogan et al.'s [14] model showed ROC AUC and PPV of 0.91 and 0.86, respectively, to the ECG models. But in contrast to Goto et al.'s [12] integrated ECG-TTE model, their sole-ECG model produced 3-4% PPV at 52-71% recalls. Regarding the CMR, the CNN-based model created by Agibetov et al. [21] showed an ROC AUC of 0.96. The ROC AUC for models with 10 and 41 variables, respectively, was 0.962 and 0.996 for the ML employed by Eckstein et al. [17]. According to reports, the 41-variable group's F1 score was 97%. A DL and a model using ML for CMR were created by Martini et al. [13], and with a 95% sensitivity and an 89% F1 score, the DL model displayed an ROC AUC of 0.982.

Furthermore, Delbarre et al.'s [15] whole-body scintigraphy (WBS)-based CNN model showed an ROC AUC of 0.999 for the five times validation group along with a specificity and sensitivity of 99.5% (± 0.4) and 98.9% (± 1.0), respectively. With a comparable ROC AUC of 0.999, their external validation group's specificity and sensitivity were 99.5% and 96.1%, respectively.

Risk of Bias Assessment

**Figure 2:**
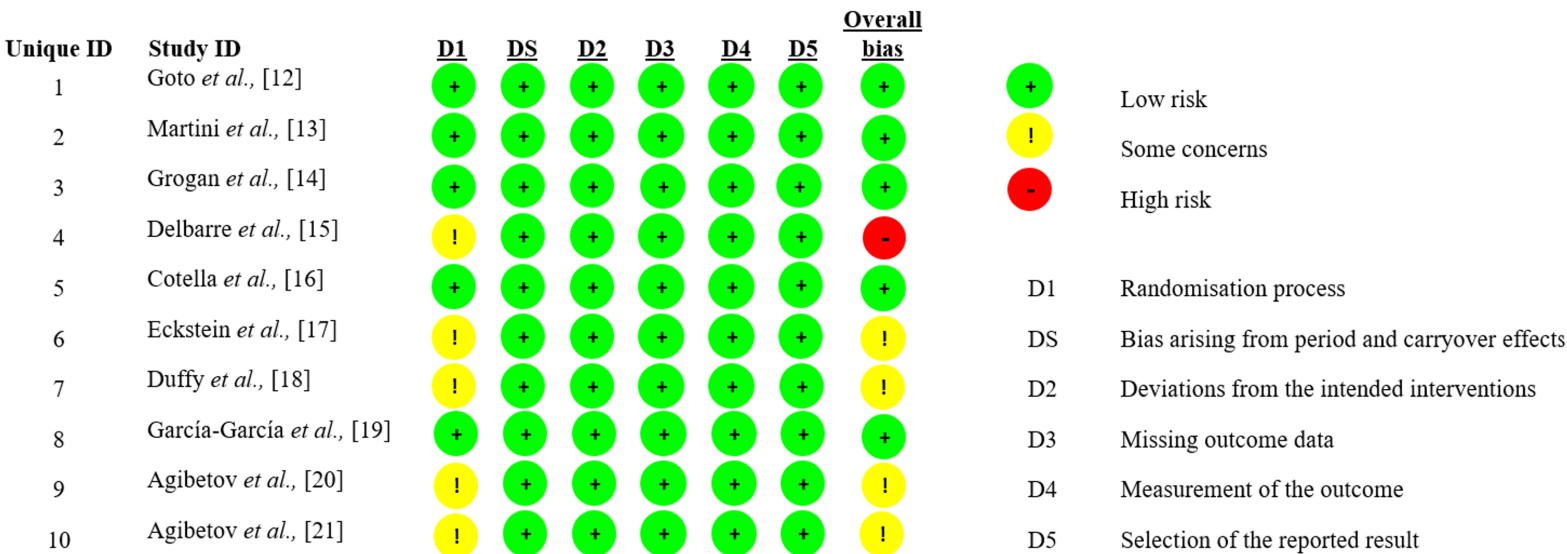
Risk of bias assessment using the QUADAS-2 tool. QUADAS-2: Quality Assessment of Diagnostic Accuracy Studies.

The risk of bias assessment showed most studies had a low risk across key domains, including randomization and blinding. Some concerns were noted in selective reporting and incomplete data, with only one high-risk domain identified [15]. Overall, the studies are methodologically reliable with minor concerns (Figure 2).

Discussion

CA is an uncommon health condition that sometimes goes undiagnosed since its symptoms might mimic those of other heart conditions [22]. Additionally, CA usually presents subtly, making early identification difficult [23]. AI may be applied as a screening method to deal with this problem [19]. A major step toward the creation of a trustworthy and precise automated diagnosis tool for CA is taken in this systematic review, where we go over a variety of ML and DL methods that can assess CMR, laboratory parameters, ECG, TTE, and bone scintigraphy.

AI and Laboratory Parameters

Using standard laboratory measurements, Agibetov et al. [21] examined the use of expert independent ML in differential diagnosis between individuals with heart failure (HF) who had or did not have an amyloid light chain (AL)/transthyretin amyloidosis (ATTR) CA relationship. Therefore, the possibility of observable differences between CA-related and CA-unrelated HF in routine laboratory testing was investigated. The medical community has a great deal of knowledge about the common forms of heart failure. On the other hand, rare HF subtypes, like those found in CA, are more likely to evade diagnosis or cause lengthy delays in the diagnostic procedure. In specialized medical facilities, rare disorders are usually identified via a drawn-out patient diagnostic procedure. Sixty-two standard laboratory values, including coagulation, blood cell count, and clinical chemistry parameters, were acquired for algorithm development in this work. Certain metrics, such as those measuring liver and renal function, were consistently measured in each patient, but other metrics showed a high level of unavailability. This study used an ML method to improve the overall forecasting efficiency by enabling non-linear prediction and increasing the precision of the baseline predictive linear model. A statistically significant association between the existence of CA and decreased risk factors for cardiovascular illnesses, specifically blood triglyceride (p = 0.008) and glucose (p = 0.008) levels, was shown by the logistic regression model. According to current research, people who develop HF as a result of CA may show symptoms of the condition even if they do not have the classic cardiovascular risk factors that are typically linked to ischemic cardiovascular disease and HFpEF [21].

Lower levels of cholinesterase and serum albumin are possible markers of impaired hepatic synthetic function in HF patients, especially when the patient's HF is more advanced at diagnosis than other HF subtypes [24]. This finding might be explained by the fact that CA is detected later than other HF subtypes. A training cohort consisting of individuals with CA along with individuals with HF but no CA (n = 415) served as the basis for the development of all the prediction models used by Agibetov et al. [20]. The performance of each prediction model was assessed using a unique prognostic validation cohort that included 124 CA-negative and 37 CA-positive patients. The most effective linear model is outperformed by the optimal model, which is built using gradient-boosted ensembles of decision trees. It is recommended to use these automated prediction techniques mainly as a means of screening for detecting possible CA patients due to the accuracy of both models. To guarantee correct diagnosis, additional confirmatory testing ought to be carried out afterward. Furthermore, the previously mentioned models for forecasting should not be applied to the general public with a low prevalence of diseases (<1%) due to the uncommon nature of CA as a kind of HF. The approach was used in groups exhibiting HF symptoms by Agibetov et al. [20]. The groups had a comparatively high frequency of amyloidosis around 23%, which is noteworthy given that CA is an uncommon condition with an incidence of one in 10,000.

The enduring importance of ML in the field of CA diagnosis is further demonstrated by another work by García-García et al. [19] that explored the same method of CA identification. The basis for developing an ML system intended to diagnose CA cases in this study was data collected from open-text clinical and nursing records obtained during hospitalization occurrences. Because the disease is persistent and there are many older individuals, finding good diagnostic techniques is essential to managing healthcare costs. Structured clinical data provides information, but it does not fully explain how a disease progresses. On the other hand, medical practitioners' unstructured writings might provide the secret to comprehending how a disease develops [25]. Because ATTR symptoms usually appear in participants 65 years of age or older, both organized and unorganized records belonging only to these subjects were included in this analysis. All hospitalized patients were taken into consideration for inclusion to guarantee a thorough evaluation of possible patients, not just those undergoing internal medicine or cardiology care [19].

Undiagnosed conditions that might have gone unnoticed could be found using this method. When applied to the group of people suffering from HF and CA illness, the algorithmic prediction produced better results. With an accuracy of 0.56 and a high precision of 0.96, this approach guarantees few false positives. Its sensitivity of 0.56, however, suggests that it might not be appropriate for a screening technique, where early detection depends on high sensitivity. The low incidence of the condition being studied can be used to justify the reduced precision and moderate F1 score [26].

It could be possible to use algorithmic analysis to identify important phrases and warning signs in smart support systems integrated into electronic health records. By using the discovered keywords in the text, these systems can offer recommendations to physicians as they are writing clinical assessments. Medical treatment can be directly impacted by this use of technology, which can also help in decision-making. Since algorithms cannot demonstrate a cause-and-effect relationship, they cannot take the position of doctors in clinical settings [19]. Nevertheless, ML algorithms can identify numerical patterns that may not be immediately obvious to humans but are derived from enormous volumes of healthcare data. An inventive method for detecting uncommon medical conditions is using medical episodes for data processing. With this method, the patient independently produces data with enough variation to be considered unique when an ML system is being trained. Improving the gathering and analysis of these multifaceted indicators may lead to the discovery of new patterns that improve patient outcomes or the efficiency of the healthcare sector [27].

AI and Transthoracic Echocardiography

To compare the fully automated AI computation of global longitudinal strain (GLS) and LVEF measures with those acquired by traditional manual methods, Cotella et al. [16] undertook a study. Using TTE, the study aimed to assess the diagnostic performance of automated AI algorithms in identifying abnormalities in patients with clinical and pre-clinical CA. Using both human and automated methods, the GLS and LVEF were measured via the apical two- and four-chamber views. The EchoGo Core 2.0 program developed by Ultromics (Oxford, UK) was specifically used for the automated measurements. The GLS and LVEF values were dichotomized using predetermined cutoff points. The thresholds were set at ejection fraction (EF) values less than 50% and GLS values more than -15.1%, respectively. The GLS and LVEF measures acquired using automated and manual methods did not differ statistically significantly, according to the statistical analysis. This was seen at the time of diagnosis as well as pre-clinical CA (p = 0.791 for LVEF and p = 0.105 for GLS, respectively). The study's findings indicate that during the pre-CA TTE, the specificity and sensitivity of AI-derived indicators for identifying aberrant LVEF were 86% and 83%, respectively. However, at the time of the CA diagnosis, these values dropped to 79% and 70%, respectively.

Furthermore, the AI-generated indices demonstrated a specificity and sensitivity of 86% and 82%, respectively, in the CA TTE concerning the diagnosis of aberrant GLS. The same indices' sensitivity and specificity were 100% and 67%, respectively, upon the diagnosis of CA. Although the exact cause of the higher specificity seen in pre-CA as opposed to CA is unknown, potential causes include varying image quality across studies, small sample sizes, the inaccessibility of ideal apical three-chamber (A3C) views for use in left ventricle GLS evaluation, and the study's retrospective design. Nonetheless, given the favorable outcomes attained, widespread use of GLS and LVEF evaluation by AI technologies may lead to a quicker assessment of different disease stages with accuracy and repeatability on par with manual techniques. According to earlier research, traditional echocardiographic measures have poor diagnostic precision for CA, mostly as a result of insufficient sensitivity.

However, cardiac deformation patterns and worldwide longitudinal strain have demonstrated greater specificity and sensitivity in the diagnosis of CA. According to Pagourelias et al. [28], even when dealing with difficult patient subgroups, the GLS/LVEF proportion is the best differentiating factor for CA. However, the results of Cotella et al. [16] show that their GLS/LVEF proportion has strong specificity (90%) but weak sensitivity (25%), indicating that it may be employed as a rule-in detection tool for CA. Furthermore, it was shown that applying the AI algorithms to these individuals will allow for the detection of CA aberrations even before the diagnosis, as required by current recommendations, by looking at two different time points. Additionally, there may be benefits to using AI to calculate GLS in the monitoring of CA patients. Cohen et al. emphasized how important GLS is while assessing CA. According to the authors, baseline GLS outperformed traditional biomarkers as a distinct indicator of survival. Moreover, better long-term survival was associated with a 2.0% drop in GLS at the 12- and 24-month follow-up following clinical intervention. These results have important clinical ramifications, especially when considering repeated echocardiograms, which are frequently conducted on patients with CA. Therefore, it is crucial to measure GLS precisely and reliably to regulate CA [29].

Duffy et al. [18] measured the thickness of the left ventricle using DL to look for signs of CA. This effort is important because it might be difficult to identify the etiological factors, such as hypertrophy, cardiomyopathy, and CA, that lead to increased ventricular wall thickness. A total of 23,745 patient records from Cedars-Sinai Medical Center were used in this investigation. For the study, parasternal long-axis and apical four-chamber echography video recordings were used. In the end, the external validation carried out in a residential environment produced an AUC for hypertrophic cardiomyopathy and CA of 0.79 and 0.89, respectively.

AI and ECG

AI models that use ECG can be a useful tool for the prompt diagnosis of CA because of their ability to concurrently examine several ECG parameters. These characteristics have been connected to the early physiological changes that take place during the illness. Furthermore, ECG abnormalities in conditions like ischemia may serve as an early warning sign before symptoms appear and structural changes are identified by TTE [24]. In 45% of cases, low voltage and pseudo infarct patterns on electrocardiography of AL CA may be indicative of the condition. Normal voltage, however, does not rule out the diagnosis.

Furthermore, low-voltage sign on ECG is only seen in 25% of patients with wild-type ATTR (ATTRwt); additionally, 20% of CA individuals with ATTRwt may exhibit hypertrophy, and ECG signals of left ventricular hypertrophy (LVH) were seen in these patients. Although they are not exclusive to either kind of CA, irregularities in the T-wave, ST-segment, and conduction system are frequently seen in both [30]. The effectiveness of a deep neural network (DNN) trained on ECG data as a tool for diagnosis to identify CA at the Mayo Clinic was investigated by Grogan et al. [14]. A total of 4995 patients, comprising both cases and controls, made up the study's sample. ECG modalities with six leads and one lead were used for the assessment of the experiment. To diagnose either form of CA, the AUC showed an estimate of 0.91 and the PPV showed a value of 0.86. Furthermore, in 59% of the patients who were part of the pre-diagnostic ECG research, which was conducted at least six months before the clinical diagnosis, the AI system was able to correctly predict the likelihood of CA. V5, the best-performing single-lead algorithm, has an AUC of 0.86 and a precision of 0.78, respectively. The performance of other single-lead methods was roughly the same. But with an AUC and accuracy of 0.90 and 0.85, respectively, the six-lead model turned out to be more successful. Smartphone-enabled sensors would be able to test for CA by allowing adaption for both six-lead ECG recordings and single-lead [14].

According to Grogan et al. [14], the DNN created may identify physiological ECG alterations unique to CA that traditional ECG analysis is unable to identify. The AI-ECG model may have the ability to suggest the identification of ATTRwt amyloidosis in females, offering further benefits in the setting of CA. Female patients are susceptible to developing ATTRwt amyloidosis and could be underdiagnosed, even though the majority of studies to date have found a male prevalence of over 90%. When traditional echocardiographic features are lacking, the AI-ECG model may be able to suggest an evaluation of ATTRwt amyloidosis.

Goto et al. [12] also used an ECG-based ML algorithm. To improve diagnostic efficiency, they also created an integrated ECG-TTE model. With a recall of 52-71%, the ECG-based AI model's PPV was discovered to be between 3% and 4%. As opposed to a PPV of 33% without pre-screening, the results show that preliminary screening with ECG greatly improved the TTE model's reliability at an average recall of 67%, leading to an enhanced PPV of 74-77%.

AI and CMR

Additionally, CMR is accepted as a diagnostic method for identifying and describing CA. CMR imaging, however, might not be enough in some circumstances to provide a reliable diagnosis of CA. At the moment, DPD (3,3-diphosphono-1,2-propanodicarboxylic acid) bone scintigraphy is an extremely relevant diagnostic test for transthyretin (TTR) CA. On the other hand, DPD bone scintigraphy is not always reliable in identifying AL CA and may often produce normal results [21]. Additionally, CMR is still a useful diagnostic tool for AL CA because it can be used to identify underlying pathological conditions like plasma cell dyscrasia, even though there may be a lack of particularity in the symptoms of AL CA on CMR scans and the sporadic occurrence of typical CMR when there is evidence of AL CA [31,32].

There is a chance that CA detection will be overlooked throughout the interpretation process in MRI sites with low referral volumes. Agibetov et al. [20] developed a fully automated approach for determining the presence of CA using CMR using a CNN. The AI model was developed using a variety of protocols, such as cardiac scans with non-contrast enhancing, improved look-locker inversion restoration, and late gadolinium enhancement (LGE). In particular, the algorithms that were trained with LGE fared better than the other algorithms used for the research.

LGE makes it possible to spot unique patterns, like the quicker removal of gadolinium from the heart and bloodstream, as compared to nonamyloid control subjects. The CNN model performed the best in this investigation, achieving a mean ROC AUC value of 0.96 and a diagnostic precision of 94% sensitivity and 90% specificity. It has been found that the AI models that can process CMR pictures exhibit the highest degree of diagnostic accuracy overall when their performance is compared across different data modalities. AI models for prediction may not require a sophisticated knowledge of CA and may not be dependent on a specific imaging methodology, as Agibetov et al. [21] showed.

To assess the precision of a DL model against an ML human reader simulation model, Martini et al. [13] compared DL and ML algorithms, taking into account all features gathered manually. In this investigation, three DL networks evaluated the LGE CMR derived from the narrow axis as well as the two and four-chamber perspectives. For the detection of CA, the results of the contrast between both ML and DL models revealed that the DL technique performed diagnostically similarly to the ML-based approach.

AI and Bone Scintigraphy

Using WBS images taken from large hospital datasets, Delbarre et al. [15] created a CNN model intending to automatically identify considerable cardiac accumulation on technetium-99m, which is defined as Perugini grade equal to or more than 2. This methodology makes it much easier to identify patients who are at elevated risk for CA. This study employed two techniques for validation.

Five equal subsets of a dataset are created to do five-fold cross-validation. Three of these subsets are then used to train the model, and the fifth is utilized for testing. Conversely, external validation is the process of evaluating a model using a different dataset, in this example, an independent hospital, that was not utilized for either the model's validation. External validation produced 99.5% specificity, 96.1% sensitivity, and an ROC AUC of 0.999, whereas five-fold cross-validation produced 98.9% sensitivity, 99.5% precision, and an AUC of 0.999. This technique has the potential to serve as a screening modality for TTR-CA given these positive results and the significant frequency of WBS conducted globally [15].

## Conclusions

The application of AI techniques in both ML and DL offers a productive way to analyze a variety of diagnostic modalities, such as laboratory values, hospitalization documentation, TTE, and bone scans. ML models that are based on open-text clinical and nursing data and standard laboratory parameters have proven useful in identifying CA. Likewise, an ML approach that employs LVEF and GLS measures derived from TTE has proven to be highly effective in identifying preliminary and symptomatic CA. In one case, it was found that the TTE model performed better in terms of diagnosis than expert cardiologists. Pre-clinical CA was detected by ECG-based models by identifying physiological ECG rhythms. Furthermore, AI models that use LGE sequences have demonstrated a high level of efficacy in diagnosing CA. All things considered, incorporating AI techniques offers a viable way to further advance CA diagnosis.

## Appendices

**Table 3:** Search strings for each database.

Database	Search string
PubMed/MEDLINE	("Artificial Intelligence"[Mesh] OR "Machine Learning"[Mesh] OR "Deep Learning"[Mesh]) AND ("Cardiac Amyloidosis"[Mesh] OR "Amyloidosis, Cardiac") AND ("Diagnosis"[Mesh] OR "Detection").
Scopus	(TITLE-ABS-KEY("Artificial Intelligence" OR "Machine Learning" OR "Deep Learning")) AND (TITLE-ABS-KEY("Cardiac Amyloidosis" OR "Amyloidosis, Cardiac")) AND (TITLE-ABS-KEY("Diagnosis" OR "Detection")).
Web of Science	(("Artificial Intelligence" OR "Machine Learning" OR "Deep Learning") AND ("Cardiac Amyloidosis" OR "Amyloidosis, Cardiac") AND ("Diagnosis" OR "Detection")) TS=(topic).
EMBASE	('artificial intelligence' OR 'machine learning' OR 'deep learning') AND ('cardiac amyloidosis' OR 'amyloidosis, cardiac') AND ('diagnosis' OR 'detection')/exp.
IEEE Xplore	("Artificial Intelligence" OR "Machine Learning" OR "Deep Learning") AND ("Cardiac Amyloidosis" OR "Amyloidosis, Cardiac") AND ("Diagnosis" OR "Detection").
